# Cancer’s Achilles’ Heel: Apoptosis and Necroptosis to the Rescue

**DOI:** 10.3390/ijms18010023

**Published:** 2016-12-23

**Authors:** Atreyi Dasgupta, Motonari Nomura, Ryan Shuck, Jason Yustein

**Affiliations:** 1 Department of Pediatrics, Texas Children’s Cancer and Hematology Centers, Baylor College of Medicine, Houston, TX 77030, USA; adasgupt@bcm.edu (A.D.); nomura@bcm.edu (M.N.); shuck@bcm.edu (R.S.); 2 Integrative Molecular and Biomedical Sciences, Baylor College of Medicine, Houston, TX 77030, USA

**Keywords:** apoptosis, necroptosis, necrosis, cancer, synergy, synthetic lethality, DNA damage, therapeutics

## Abstract

Apoptosis, and the more recently discovered necroptosis, are two avenues of programmed cell death. Cancer cells survive by evading these two programs, driven by oncogenes and tumor suppressor genes. While traditional therapy using small molecular inhibitors and chemotherapy are continuously being utilized, a new and exciting approach is actively underway by identifying and using synergistic relationship between driver and rescue genes in a cancer cell. Through these synthetic lethal relationships, we are gaining tremendous insights into tumor vulnerabilities and specific molecular avenues for induction of programmed cell death. In this review, we briefly discuss the two cell death processes and cite examples of such synergistic manipulations for therapeutic purposes.

## 1. Introduction

Apoptosis, a programmed cell death, is one of the most published areas of cell biology. However, it has yet to be completely understood, even with a wealth of new information that continuously enriches the field, but also challenges older notions. Apoptosis plays a crucial role in maintaining normal physiological states, such as tissue homeostasis, and its deregulation contributes to pathological states, such as neurodegenerative diseases, autoimmunity, and cancer [1,2]. Thus, having a better understanding of this complex biological process can lead us to more nuanced therapeutic interventions in order to counter the diseased state. A second regulated cell death mechanism, necroptosis, has generated lot of interest in the last several years [3,4,5]. Necroptosis shares some of the features of apoptosis, while owning some unique characteristics itself. Another common form of cell death, necrosis, occurs in response to trauma or injury. In recent times, there have been indications that necrosis might also be a process of programmed cell death. For this review, we will discuss the background of apoptosis and necroptosis, types of apoptosis, differences between apoptosis and necroptosis, as well as the effector molecules and pathways involved in these two methods of programmed cell death. Finally, we will focus on how certain intrinsic genotypic features of cancer cells can help the cell to escape apoptosis and necroptosis, but that can be exploited to induce synergism leading to synthetic lethality for the development of novel therapeutic strategies to treat malignant conditions.

## 2. Apoptosis

### 2.1. What Is Apoptosis?

The word apoptosis owes its origin to a Greek word meaning “falling off the tree” [6]. Apoptosis is a tightly regulated process that can occur during normal physiological processes as well as during pathologic conditions. For example, during embryogenesis, aging and healing, apoptosis is required to maintain proper homeostasis within tissue. It can also serve several embryonic development purposes. For example, during the development of the vertebrate nervous system, over 50% of the nerve cells die. In addition, cells in the intestine and bone marrow have high turnover rates and undergo apoptosis frequently [7]. In addition, apoptosis serves to eliminate potentially harmful mutated cells, and failure to do so could lead to uncontrolled cell growth, which ultimately ends in cell transformation and tumor development.

Apoptosis has characteristic features of membrane blebbing, cell shrinkage, nuclear condensation (pyknosis), DNA fragmentation, and finally, karyorrhexis or fragmentation of the nucleus, followed by formation of apoptotic bodies. These resultant apoptotic bodies end up being phagocytosed by neighboring cells, such as the macrophages, parenchymal cells, or neoplastic cells [8,9,10]. Apoptosis may be activated by several factors, known as initiators, which include ultraviolet radiation, energy depletion, drugs, deprivation of growth factors, etc.

### 2.2. The Main Players of Apoptosis: Caspases

Apoptosis is a highly conserved process that uses proteolytic enzymes, known as caspases, as main contributors towards execution of its cellular functions [11]. The caspases are initially made in inactive precursor forms ranging from 30–50 kDa, and become active by proteases or through autocatalysis [12]. These enzymes generally consist of three domains, including an amino terminal domain, a large subunit and a small subunit. The proteolytic cleavage of the two subunits leads to activation of the caspases. The two units then heterodimerize to form the functional catalytic enzyme [12,13]. Caspases that are specifically involved in apoptosis can be divided into two broad categories. The initiator caspases consist of caspase-2, -8, -9, and -10 (found only in human), which perform upstream functions in the cellular pathway. Their activation is generally required for activation of the second set of caspases, often referred to as the executioner caspases. The executioner caspases include caspase-3, -6, and -7 [14].

### 2.3. Types of Apoptosis

**Figure 1:**
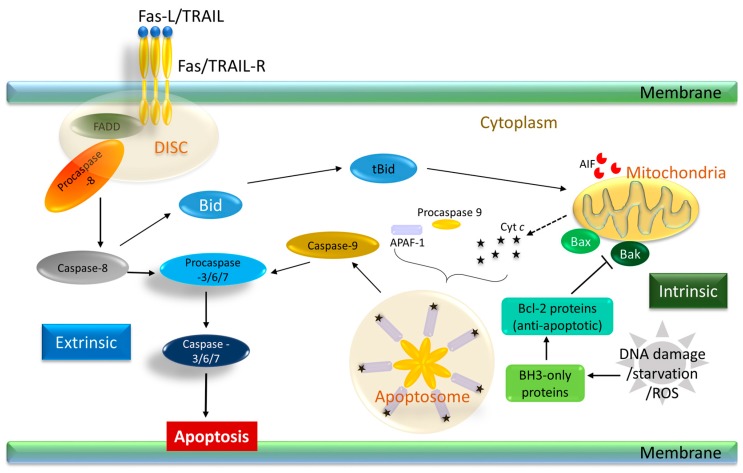
Representative apoptosis pathway and associated factors. The two pathways, namely intrinsic and extrinsic pathway, functions via mitochondria and death receptors, respectively, as shown here. In the extrinsic pathway, death ligands, such as Fas-L or TRAIL, bind to their specific receptors to initiate the formation of the DISC complex, resulting in activation of caspase-8 and subsequent activation of the effector caspases-3/6/7. In the extrinsic pathway, procaspase-9 binds to cytochrome *c* bound APAF-1, forming the apoptosome complex. The truncated and activated form of Bid is indicated as tBid here. Arrows indicate induction of apoptosis. Blunt arrows indicate inhibition of apoptosis. Dashed arrows are used to show cytochrome *c* release from mitochondria. Fas-L, CD95-ligand; TRAIL, tumor necrosis factor (TNF)-related apoptosis-inducing ligand; TRAIL-R, TRAIL receptors; FADD, Fas associated death domain; DISC, death inducing signaling complex; Bcl-2, B-cell lymphoma 2; BH, Bcl-2 homology; AIF, apoptosis-inducing factor; ROS, reactive oxygen species.

Apoptosis can be fundamentally divided into two paths: the extrinsic and the intrinsic, as summarized in Figure 1. The extrinsic pathway receives and processes extracellular death-inducing signals that work through death receptors on cell surfaces. In contrast, the intrinsic pathway receives and integrates intracellular signals and functions through the mitochondria. In the extrinsic pathway, extracellular signals or stress prompt ligands, such as the tumor necrosis factor (TNF), CD95-ligand (CD95-L or Fas-L), TNF-related apoptosis-inducing ligand (TRAIL or Apo2-L), and TNF-like ligand 1A (TL1A), to bind to the death receptors. Death receptors are generally members of the tumor necrosis family (TNF), such as the TNF receptor-1 (TNFR-1), Fas, Apo-1, and TNF-related apoptosis-inducing ligand receptors (TRAIL-R). This receptor–ligand binding leads to the recruitment of the procaspase-8 enzyme to the death inducing signaling complex (DISC). At the cytoplasmic end of the death receptor, adaptor proteins, such as the Fas associated death domain (FADD), or TNFR-associated death domain (TRADD), are recruited. This results in the dimerization and activation of caspase-10 and caspase-8 monomers and finally to the dimerization of caspase-8 [15]. In some cells, known as type I cells, this step leads to the direct activation of the executioner caspases, caspases-3 and -7, leading to cell death. On the other hand, in type II cells, the intrinsic pathway needs to be activated before apoptosis is initiated. Whether a cell would take the type I or II route depends on the presence of inhibitor of apoptosis proteins (IAPs), which block the executioner caspases. X-linked inhibitor of apoptosis (XIAP) binds directly to caspase-9, -7 and -3 and inhibits their activation. The cellular IAP (cIAP) proteins, which are ubiquitin ligases, can inhibit caspase activity indirectly, resulting in elicitation of pro-survival signaling. Proteins released from the mitochondria, such as the second mitochondrial protein Second mitochondria-derived activator of caspases (SMAC)/DIABLO, can suppress the IAPs and eventually release the block [16,17,18].

Intracellular signals, such as DNA damage, growth factor depletion, hypoxia, accumulation of unfolded proteins, or cytotoxic drugs, can initiate the intrinsic, or mitochondrial apoptotic pathway. In this pathway, caspase-9 is the initiator caspase, while the B-cell lymphoma 2 (Bcl-2) family of proteins function as the main regulators. The Bcl-2 proteins are broadly categorized by their Bcl-2 homology (BH) domain consisting of the effector pro-apoptotic members, which have the BH1-BH3 domains, such as Bak, Bax, and Bok. Members with BH1-BH4 domains are the anti-apoptotic proteins, such as Bcl-2, MCL-1, and Bcl-_X_L. These proteins bind to the pro-apoptotic members, rendering them inactive. A third set of proteins contain only the BH3 domain, such as Bim, Bid, Puma, Noxa, and Bad. These proteins are responsible for inhibiting the anti-apoptotic Bcl-2 proteins, thereby releasing the repression and inducing disruptions in the outer mitochondrial membrane, eventually promoting mitochondrial outer membrane permeabilization (MOMP) [19,20,21,22]. In times of growth factor deficiencies, such as low levels of interleukin-3 in lymphocytes or insulin-like growth factor 1/2 in epithelial cells, apoptosis can be initiated through Bim. On the other hand, Noxa and Puma are involved in apoptosis that is initiated during TP53-driven DNA damage response when there is significant DNA damage and genomic instability [23]. The level of the pro- and anti-apoptotic molecules determine activation of the effector proteins: Bak and Bax. Activation of the effector proteins causes MOMP, which results in the diffusion of proteins that generally reside between the outer and inner mitochondrial membrane to the cytosol. One of these proteins is cytochrome c. Binding of cytochrome c to the adaptor protein, apoptotic protease-activating factor-1 (APAF-1) leads to a series of conformational changes of APAF-1, and ultimately the recruitment and activation of caspase-9 (Figure 1). This complex is known as the apoptosome (cytochrome c, caspase-9, and APAF-1) [24]. Activation of caspases-9 leads to activation of caspase-3, -6/7 which then cleave several cellular targets such as poly ADP-ribose polymerase (PARP), inhibitor of caspase activated DNAse and lamin. These biochemical reactions ultimately lead to cellular denaturation and death [25]. Additionally, it should be noted that there is existence of crosstalk between the extrinsic and intrinsic pathways: when the death receptor stimulated DISC formation (as described under extrinsic pathway) is not sufficient, caspase-8 mediated cleavage of Bid (tBid) can activate Bak/Bax resulting in amplification of the death signal [26,27,28].

Caspase independent Apoptosis: One of the first proteins that was found to mediate caspase independent cell death was the Apoptosis-inducing factor (AIF). AIF is involved in apoptosis only in certain cells. It is anchored to the inner mitochondrial membrane (IMM) and later gets released into the cytosol [29,30]. AIF acts as a nicotinamide adenine dinucleotide hydride (NADH) oxidase and is proposed to play a role in oxidative phosphorylation [31]. When stimulated by certain apoptotic triggers, AIF is cleaved from the IMM to shorter fragment. This fragment can then be released in the cytosol. Once in the cytosol, it can translocate to the nucleus and take part in chromatin condensation and DNA fragmentation. The mechanism by which AIF can exert these functions is not very well understood and is an active area of research.

Mitotic Catastrophe and Apoptosis: Mitotic catastrophe is a type of cell death that occurs during mitosis or resulting from mitotic failure. It is thought to result from a deficiency in DNA repair and spindle assembly checkpoints after cellular damage. Due to the error in DNA, failure to arrest the cells before they enter mitosis results in aberrant chromosomal segregation. This finally leads to the activation of caspase-2 and/or mitochondrial membrane permeabilization with the release of cytochrome c, which ultimately leads to apoptosis. The fractions of cells that fail to induce apoptosis will undergo asymmetric division, resulting in aneuploidy. The main players in mitotic catastrophe include cell cycle kinases, such as cyclin B1 dependent kinase CDK1, Aurora kinase, p53, Survivin, and Bcl-2 family members [32].

### 2.4. Apoptosis and Cancer

Hanahan and Weinberg, in their seminal review [23,33], pointed out that one of the hallmarks of cancer is the ability to escape apoptosis. In recent years, a heightened interest has been shown to understand underlying mechanisms of apoptosis to strategize therapeutic interventions. Many of the characteristics displayed by cancer cells, such as inactivation of cell cycle checkpoints, would normally induce apoptosis in healthy cells [34]. The first indication of the involvement of the apoptotic machinery in tumorigenesis came from the identification of the *bcl-2* oncogene at the chromosomal breakpoint of *t*(14;18) translocation that was found in non-Hodgkin’s Lymphomas. The translocation brought the gene under the control of the immunoglobulin heavy chain enhancer, resulting in dysregulation of *bcl-2* expression [35,36]. Later, *bcl-2* gene amplification was also found in follicular lymphoma and small cell lung cancer [35,37], and eventually, about 50% of human cancers have been shown to have an elevated expression of Bcl-2 [38]. Besides Bcl-2, other members of the apoptotic pathways, such as Bcl-_X_L have been found to be oncogenic drivers in colorectal cancer, while Bax, yet another member, has been inactivated in some other cancers, such as colon cancer and hematopoietic malignancies [39,40]. Contrary to this data, studies have demonstrated that overexpression of Bcl-2 (anti-apoptotic) in hepatocytes or loss of Bid (pro-apoptotic) in liver carcinogenesis can actually be inhibitive to tumor progression [41,42]. Although seemingly counterintuitive, dying cells can secrete stimulatory factors that have growth promoting effects on surrounding cells. In addition, cancer cells compete for nutrition and space; in the event of cell death, an empty space is left, and the more aggressive clones can take over that space [43]. What is becoming clearer, though, is that apoptosis might no longer be a water tight barrier against tumorigenesis as was previously thought.

## 3. Necroptosis

### 3.1. Genesis of a Novel Concept “Necroptosis”

Death stimuli, and the endogenous expression level of death signaling effectors, determine the route of cell death. Among the different mechanisms of cell death, there is much more information on apoptosis than necrosis, pyroptosis, or autophagy. As described above, apoptosis is a well-known form of programmed cell death induced by the activation of caspase-8 or caspase-9. Like apoptosis, cell death by pyroptosis is also a form of caspase-dependent cell death, but involves different death stimuli. Furthermore, contrary to apoptosis, in pyroptosis, caspase-1 is activated by the formation of inflammasome complex as an antimicrobial response during pathogenic infection of *Salmonella* and *Shigella* species [44]. In addition, another mechanism of cell death has recently been identified that is morphologically necrotic, but is induced by the same stimuli as apoptosis. This programmed necrotic cell death, which is referred to as necroptosis, is thought to be induced by apoptotic death stimuli, such as TNF-α and Fas ligand.

The signal transduction pathway for necroptosis is reported to be caspase-independent. Classically, when a ligand binds to the death receptor of the cell, receptor interacting protein kinase 1 (RIP1) is deubiquitinated by Cylindromatosis (CYLD). RIP1 can then freely migrate to the cytoplasm and form a complex with receptor interacting protein kinase 3 (RIP3), FADD, caspase-8, and TRADD. Most human cells express caspase-8 in an activated form, which suppresses RIP1 or RIP3. However, if the endogenous expression of caspase-8 is absent or silenced, RIP1 remains activated via phosphorylation of serine 161 [5,45,46,47,48,49,50]. Cellular FLICE-like inhibitory protein (cFLIP) plays one of the most important roles in preventing caspase-8 activation as an anti-apoptotic regulator in cancer cells. CFLIP has mainly two isoforms, long isoform (cFLIPL) and short isoform (cFLIPS), and both of them can function as anti-apoptotic proteins. Thus, cFLIP has been thought to be a pro-necroptotic protein. Interestingly, however, while cFLIPS promotes necroptosis, cFLIPL can be anti-necroptotic since it has a caspase-like domain [51]. A complex consisting of RIP1, RIP3, FADD, and TRADD can induce reactive oxygen species (ROS) production, followed by plasma membrane permeabilization, and cytosolic ATP reduction, which induces necroptosis [5,52]. More recently, mixed lineage kinase domain-like protein (MLKL) was reported to be essential towards triggering necroptosis by stepwise phosphorylation of RIP1-RIP3-MLKL complex, also called the necrosome. RIP1 kinase phosphorylates RIP3, followed by MLKL phosphorylation and homo-trimerization. This trimerized MLKL translocates to the plasma membrane and induces necrotic plasma membrane permeabilization, which is one of the executioners of necroptosis [53]. Necrosis has been extensively studied since the early ages of medicine, and some patterns of necrosis have been shown to be programmed, as observed in apoptosis. Therefore, the concept of necroptosis is a significant paradigm shift from the previous notions of cell death.

### 3.2. Regulation of Necroptosis Machinery

**Figure 2:**
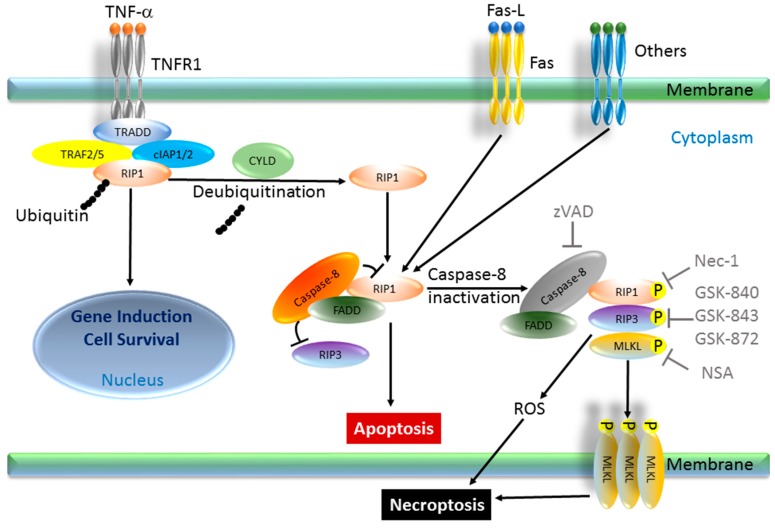
Representative necroptosis pathway and associated factors. In a TNF-α-induced necroptosis model, TNFR1 is stimulated by TNF-α and engages a complex formation consisting of TRADD, RIP1, TRAF2 (or TRAF5), cIAP1, and cIAP2, with subsequent polyubiquitination of RIP1. This pathway leads to gene induction and cell survival. However, once CYLD targets RIP1 for de-ubiquitination, another cytosolic complex consisting of caspase-8, FADD, RIP1 and RIP3 is formed. In this complex, an activated form of caspase-8 cleaves and suppresses the activity of RIP1 and RIP3, thereby blocking necroptosis and promoting apoptosis. When the cleavage of RIP1 and RIP3 is blocked by caspase-8 inhibitors, such as zVAD, or by silencing of caspase-8, a third complex, named necrosome, consisting of RIP1, RIP3, and MLKL, is formed. Mutually direct or indirect phosphorylation of these components in the necrosome initiates necroptosis by bursts of ROS from the mitochondria or by forming homotrimer of MLKL, leading to necroptosis. Necroptosis can be inhibited by Nec-1 (RIP1 inhibitor), GSK-840/-843/-872 (RIP3 inhibitor), or by NSA (MLKL inhibitor). TNFR, TNF receptor; TRADD, TNFR-associated death domain; TRAF, TNFR-associated factor; RIP, protein kinase; cIAP, cellular inhibitor of apoptosis proteins; CYLD, Cylindromatosis; MLKL, mixed lineage kinase domain-like protein; zVAD, z-Val-Ala-Asp; GSK; NSA, necrosulfonamide; P, Phosphorylation.

Necroptosis is inducible in many types of cells if apoptotic death signaling is inhibited by pretreatment with z-VAD-FMK and cycloheximide prior to exposure to death ligands, such as TNF-α and the Fas ligand. The resulting morphological features are similar to those of necrosis [5,45]. In the ischemic brain, this type of cell death tends to be active. Necrostatin-1 (Nec-1) was first reported to be the agent that suppresses ischemic brain injury in mice through a mechanism that is distinct from apoptosis [54]. Necrostatin-1 was later discovered to be a specific inhibitor of RIP1 [55]. Therefore, cell death that is rescued by Necrostatin-1 can be considered to be necroptosis, and RIP1 is believed to be the key factor for necroptosis. In addition to RIP1, RIP3 is also essential for necroptosis, as RIP3 is regulated by the caspase-8-FLIP complex [56] and mediates the embryonic lethality of caspase-8-deficient animals [57]. Moreover, RIP3-knockout mice are quite vulnerable to some viruses [48]. Thus, necroptosis plays an important role in the inflammatory response or innate immune response to viral infection. After the discovery of Necrostatin-1, several other chemical compounds that can pharmacologically inhibit necroptosis were discovered, contributing to furthering the understanding of the mechanism of necroptosis, such as GSK-843, GSK-872, GSK-840 (RIP3 inhibitor), and necrosulfonamide (NSA; MLKL inhibitor) [3,58,59] (Figure 2).

### 3.3. Downstream and Upstream of Necroptosis Pathway

The mechanism of necroptosis induced by the representative stimulators, namely TNF-α and Fas ligand, have been well studied. In addition to TNF-α and Fas ligand, other agents, such as kuguaglycoside C, a constituent of *Momordica charantia* [60] and Hemagglutinating virus of Japan-envelope (HVJ-E), an inactivated form of Hemagglutinating virus of Japan, have been reported to stimulate necroptosis [61]. The key executors of necroptosis are RIP1 and RIP3, the downstream factors of these kinases being ROS, MLKL, and AIF. AIF is a Janus protein that exerts redox activity in the mitochondria and pro-apoptotic activity in the nucleus, but can also regulate necroptosis [62]. ROS also regulates apoptosis through other mechanisms involving AIF. Thus, absolute markers of necroptosis that exist downstream of RIP1 and RIP3 have yet to be discovered.

Following death receptor stimulation, the upstream actions of the necroptotic signaling, i.e., the mechanism by which RIP1 or RIP3 are phosphorylated, have never been elucidated. However, it has been recently reported that calcium-calmodulin-dependent protein kinase II (CaMK II) can phosphorylate RIP1 in several neuroblastoma cell lines whose expression of caspase-8 is silenced, although it was not clear whether this regulation of RIP1 by CaMK II was direct or indirect [61]. Another group reported that CaMK II can serve as the substrate for RIP3, inducing necroptosis in a myocardial ischemia model [63]. These findings imply that CaMK II is one of the key factors to directly trigger necroptosis. CaMK II is activated through the binding of calcium and calmodulin after the increase in cytosolic calcium, possibly triggering apoptosis via the ER stress stimulation or other pathways. Thus, necroptotic cell death signaling via CaMK II activation may only be encountered under limited conditions.

## 4. Difference between Apoptosis and Necroptosis

### 4.1. Morphological Findings

A number of features discovered over the past several years, especially those involved in necroptosis, have the potential to further illustrate the differences between apoptosis and necroptosis. Classically, several cell morphological changes may be detected using transmission electron microscope. As discussed previously, membrane blebbing, cytoplasmic shrinkage, formation of apoptotic body, nuclear fragmentation, and chromatin condensation are typical indicators of apoptosis. Conversely, membrane permeabilization and swelling of intracellular organelle or mitochondria are specific characteristics of necroptosis. These morphological differences are very useful and critical. However, use of transmission electron microscopy as a means of detection is time-consuming, so alternative methods to evaluate these features of cell death are needed.

### 4.2. Functional Findings

In order to compare intracellular function, evaluating the activity of caspase-3 and/or caspase-7 by Western blotting or by caspase-3/7 enzymatic assay can be useful for identifying apoptosis. Activation of RIP1, RIP3, and MLKL, which are specific for necroptosis, may be detected by Western blotting or by labeling with radioisotope 32P. Necrosome formation, which is also specific for necroptosis, can be revealed by proximity ligation assay or immunoprecipitation, followed by Western blotting. In addition, ROS production can be a marker for necroptosis, but this may be induced in some situations of apoptosis as well. Therefore, aside from ROS burst, intracellular ATP increase, a feature specific of apoptosis, and ATP deletion, a characteristic of necroptosis, can be good markers to distinguish each event, while both can be measured by luciferase assay. In addition, pretreating the target cells with caspase inhibitor zVAD, RIP1 inhibitor Nec-1, RIP3 inhibitor GSK-840/-843/-872, and/or MLKL inhibitor NSA can be a method of comparison between apoptosis and necroptosis.

Taken together, a multimodal approach using these tools can elucidate the mechanism of cell death and clarify the morphological and functional differences of apoptosis and necroptosis (Table 1).

## 5. Exploiting Apoptosis and Necroptosis for Therapeutic Development

### 5.1. Synergism and Synthetic Lethality: Inducing Apoptosis in Cancer

One of the pitfalls of chemotherapeutic drugs is that they target normal cells, especially rapidly dividing ones such as bone marrow, hematopoietic and intestinal mucosal cells. Some drugs can also target cells that are not necessarily dividing at a rapid rate, as shown by the side effects of doxorubicin on the heart. Thus, it is necessary to develop drugs that can selectively kill cancer cells, while limiting their cytotoxic effects on normal cells. This might most effectively be achieved by exploiting and targeting the genotypic aberrations specific to cancer cells. Understanding and exploiting these aberrant pathways that drive cancer cell growth can serve to induce apoptosis or necroptosis.

**Figure 3:**
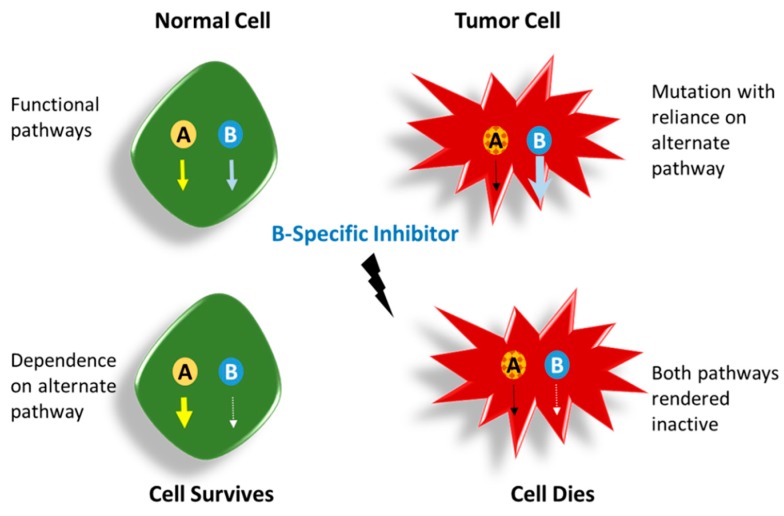
Schematic depicting the basic mechanism of induction of synthetic lethality by an inhibitor. Genes A and B are in a synergistic relationship. In a normal cell, when one pathway is obliterated, the cell heavily relies on the other pathway and survives. In the cancer cell, however, one pathway is already dysregulated, aiding in malignancy. Targeting the other pathway will lead to selective cell death. Thick arrow indicates more dependence while thin arrow indicates less dependence.

To this end, mutations in oncogenes, which ultimately drive tumorigenesis, can be exploited to selectively kill the tumor cells themselves by synthetic lethality, leading to synergistic apoptosis. As depicted in Figure 3, this approach consists of identifying sets of genes, usually two, that would be in a synthetic lethal relationship. The inhibition or elimination of any of these genes confers a survival advantage, but elimination of both genes would kill the cell [64]. Killing the tumor cells, while sparing the healthy ones, makes this a very attractive therapeutic approach. In recent times, genes specifically involved in the DNA repair pathway have emerged as viable targets for this purpose. Other than driver oncogenes, tumor suppressor genes also play a major role in tumorigenesis. Targeting tumor suppressor genes has been conceptually challenging due to their mode of action, but using the approach of synthetic lethality has the potential to overcome many of these challenges. Since the first studies in *Drosophila melanogaster* [65], synthetic lethality has been explored quite extensively in human and other models and methods using this principle are being developed rapidly [66,67,68]. In the next section, we outline some of the relevant genes/proteins and/or pathways that have been studied in the context of synthetic lethality.

#### A. Poly (ADP-Ribose) Polymerase-1 (PARP1) and Breast Cancer Susceptibility Gene1/2 (BRCA1/2)

**Figure 4:**
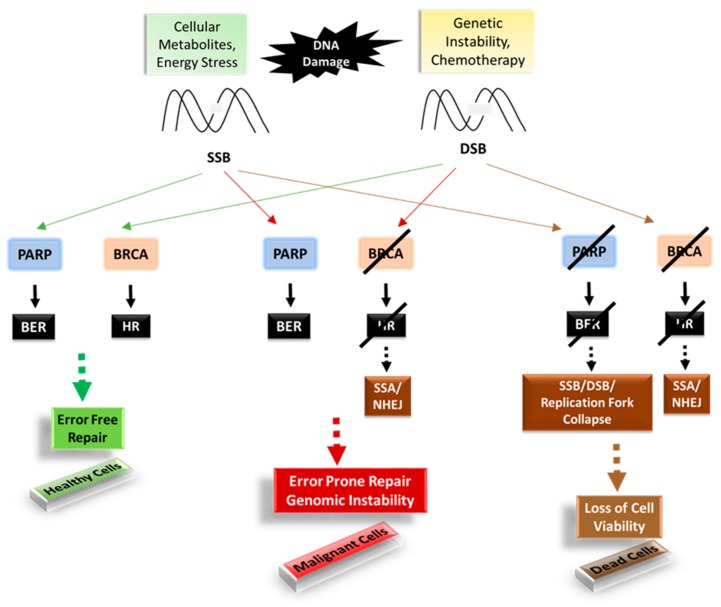
Schematic showing the synergistic relationship between PARP inhibitors and BRCA1/2. SSB, Single strand DNA breaks; DSB, double strand breaks; PARP, poly ADP ribose polymerase; BRCA, breast cancer susceptibility gene; BER, base excision repair; HR, homologous repair; SSA, single strand annealing; NHEJ, non-homologous end joining. Solid arrows indicate the pathway the proteins are involved while dashed arrows indicate the result of them.

Perhaps the first prominent example, studied for the purpose of inducing synthetic lethality, are the BRCA1 and BRCA2 genes that are involved in the DNA repair pathway [69,70]. Single strand DNA breaks (SSBs) are the most common DNA damage that occurs as a result of intracellular metabolites or spontaneous DNA decay. In healthy cells, damage can be repaired through base excision repair by PARP1 prior to entry into the S phase. If PARP is inhibited, the cells would enter S phase with the SSB, resulting in collapse of the replication fork and, ultimately, double strand breaks (DSBs). BRCA1 and 2 play a major role in protecting cells from DSBs. BRCA1 has a broader role, and other than DNA repair, is also involved in transcriptional regulation and chromatin remodeling. BRCA2, on the other hand, has been found to be predominantly involved in DSB repair by directly binding to RAD51 recombinase, an enzyme required for DSB repair by error-free homologous repair (HR) [71]. Cells that lack functional BRCA1/2 try to counter these DNA lesions by non-canonical, error-prone, unstable mechanisms, such as single strand annealing (SSA) or non-homologous end joining (NHEJ), resulting in overall genomic instability and hereditary predisposition towards malignancy [72]. Mutations in these genes are a high risk factor for breast cancer, ovarian cancer, and other cancers [73,74]. Thus, for these genes, inhibiting PARP would cause synthetic lethality leading to cell death (Figure 4).

As a result, PARP inhibitors have been widely used in pre-clinical and clinical settings [75] and phase 2 results with Iniparib (Sanofi-Aventis) were impressive in triple negative breast cancers (TNBC), an aggressive form of breast cancer. Another PARP inhibitor, Olaparib (AZD-2281, AstraZeneca), showed response in 41% of women with BRCA1- and BRCA2-deficient breast cancer in a phase 2 trial. Olaparib was the first PARP inhibitor that was approved as maintenance therapy for responding patients [75,76]. These were followed by reports from several other trials in BRCA1- and BRCA2-deficient breast cancer, ovarian cancer, melanoma, colorectal cancer, hepatocellular carcinoma, and cervical cancer. Although effective in most cases, over the years, data from these trials has shown that restoration of the HR (by a second mutation in an already mutated BRCA genes), or modulation of PARP itself, can lead to resistance. Thus, a deeper understanding of PARP inhibitors and screening for proper genotypic background is required for effective treatment and in order to avoid resistance [77].

#### B. RAS/RAF and MEK Pathway

One of the most mutated oncogene in human cancer happens to be *kras* [78]. Activation of KRAS leads to further activation of downstream intracellular signaling through mitogen-activated protein kinase (MEK) and extracellular signal-regulated kinase (ERK). Inhibiting MEK alone has largely been cytostatic and resulted in very little apoptosis. Thus, even though it was inhibitory to tumor growth, it was not successful in effective tumor regression [79]. In addition, resistance to rapidly accelerated fibrosarcoma (RAF)- and MEK-targeted therapy has been a major challenge [80,81] due to acquired resistance in some patients. Some of these resistances are due to BRAF gene mutation and those with rat sarcoma (RAS) mutation remain largely insensitive [82,83,84,85]. Lin and colleagues, through a genetic screening, have shown that an alternate escape pathway is responsible for the resistance. Hippo pathway effector, Yes-associated protein (YAP), is the major player in this alternate pathway [86]. Accordingly, those with BRAF-mutant tumors can be treated by simultaneously inhibiting YAP and RAF or MEK, resulting in synthetic lethality in several BRAF-mutant tumors as well as in RAS-mutant tumors.

In the case of non-small cell lung cancers (NSCLC), Huang et al. used a synthetic lethal screen to induce apoptosis in *kras* mutant cells [87]. In these cells, activation of RAF, MEK, and ERK ultimately leads to c-MYC activation, which in turn inhibits the expression of cFLIP and can sensitize the cells to TRAIL-mediated apoptosis by modulating the expression of TRAIL receptors. On the other hand, Ras activation can upregulate the apoptotic inhibitor XIAP through the AKT pathway. The activation of XIAP can be overcome by second mitochondria-derived activator of caspases (SMAC), which can bind to IAPs, thus freeing the caspases. These interactions can be exploited specifically in KRAS-activated premalignant lung cells by allowing TRAIL to sensitize the cells to apoptosis and using small molecule SMAC mimics.

In a more recent study, Lambo and colleagues used a siRNA and shRNA screen against a cohort of colorectal cancer and lung cancer cells with *kras* mutations. They found that resistance to MEK inhibitors resulted from RAS activation and sustained MEK-ERK signaling. This can be reversed by inhibiting MEK and targeting the RAF kinases, resulting in induction of apoptosis in these cells [88].

In another study, it was reported that when MEK is inhibited, there is an increase in the pro-apoptotic protein, Bim. However, this induction was insufficient to cause apoptosis on its own, as the anti-apoptotic protein, Bcl-_X_L, from the BH3 family, bound and repressed BIM. Thus, when Bcl-_X_L was inhibited to bind and repress Bim, in conjunction with direct MEK inhibition, it led to significant apoptosis in many KRAS mutant cell lines from varying origins and caused tumor regression [89].

#### C. MK2, ATM/ATR and Chk1/2 Kinases

Cells with mutation-induced genotoxic stress have chronic activation of the DNA damage response pathway, resulting in cell cycle arrest and DNA repair. The two major pathways in DDR operate as follows: One is under the control of ataxia-telangiectasia mutated (ATM) and Chk2 that is activated under stress caused by double strand breaks (DSB), and the other, under ataxia-telangiectasia and rad-3 related (ATR) and Chk1, is activated by UV-induced DNA damage or during replication fork collapse in S phase [90,91,92]. In the background of wild type p53, when activated, these pathways work towards inactivating Cdc25A/B/C via p21, which leads to a temporal cell cycle arrest in G1/S in order to initiate DNA repair [93,94]. In healthy cells, if there is damage beyond repair, then as a protective measure, apoptosis is induced via a p53 mediated pathway [95]. In the absence of wild type p53, which can be found in many tumor cells, a parallel, recently studied pathway involving p38 dependent MAP kinase-activated protein kinase 2 (MK2) has been reported to be recruited in the ATM/ATR network [96], resulting in cell cycle arrest. The p38/MAPK/MK2 pathway is activated to arrest cells at G2/M or intra-S, following DNA damage. As part of this genotoxic stress response pathway, the MK2/p38a complex is activated. Thus, it can be hypothesized that chemotherapeutic inhibition of MK2 in p53 deficient cells would result in cells undergoing mitotic catastrophe that results in cell death [96]. Indeed, based on this, Dietlein and colleagues [97] found that tumors with oncogenic *kras* have simultaneous mutations in p53 and other cell cycle regulating factors, rendering them addicted to Chk1/MK2 mediated checkpoint repairs. They further observed that, in these tumors, driven by KRAS or BRAF mutations, when Chk1 and MK2 were individually inhibited, no cytotoxic effect was found. However, when a combined inhibition was done, a strong synergistic effect leading to apoptosis was observed.

#### D. Avian Myelocytomatosis Viral Oncogene Cellular Homolog (MYC)

*c-myc* is one of the most prominent oncogenes frequently found to be deregulated in many cancers, making it a very attractive candidate for development of cancer therapeutics. Currently, no therapeutic approach is available to directly target the oncogenic activity of MYC. A growing body of literature shows that several oncogenes, coupled with MYC deregulation, can be the primary factor of tumorigenesis and targeting one of these partners can bring about synthetic lethality. In as early as 2004, Wang and colleagues demonstrated [98] that overexpression of MYC can render cells sensitive to TRAIL and Death Receptor 5 (DR5) agonists that lead to increased apoptosis. This leads to the hypothesis that MYC-mediated synthetic lethality can be induced by targeting other partner oncogenes in the MYC overexpressing cells with very little cytotoxic effect to the healthy cells.

Goga and colleagues [99] found that cells that overexpress MYC can be led to apoptosis by small molecule inhibitors that target CDK1. CDK1 binds to cyclin B and is essential for G2-M transition. By inhibiting CDK1, cells arrest in G2 in normal cells, whereas, in transformed cells overexpressing MYC, results in apoptosis. Furthermore, cells that harbor high MYC activity, along with high RAS activity, showed even more apoptotic induction when CDK1 was inhibited. Overexpression of MYC primes a cell towards apoptosis by increasing Bcl-2 family members with BH3-only domain (described earlier). In this setting, when CDK inhibitors were used, degradation of Survivin ensued, which normally remains stable via CDK1 phosphorylation. Survivin is an inhibitor of apoptosis (IAP) protein, which can modulate apoptosis by preventing activation of caspases.

Blocking CDK1 in TNBC cells was also highly effective by synergistically inducing apoptosis, as reported by Horiuchi and colleagues [100]. TNBC has long been a clinical challenge for the lack of targetable molecules, as they are negative for the estrogen, progesterone, or HER2 receptors. In this study, the authors reported that high MYC expression and increased MYC signaling was associated with poor prognosis in TNBCs. They found in these tumors that inhibiting CDK resulted in upregulation of the pro-apoptotic Bcl-2 family member, Bim, which conferred synthetic lethality, resulting in tumor regression.

MYC’s oncogenic properties include high cell proliferation and growth, which is related to high protein synthesis. Using a transgenic mouse system, Pourdehnad and colleagues reported an interesting convergence of MYC and mammalian target of rapamycin (mTOR) pathway [101]. Their results show that one of the mTOR substrates, 4EBP1 (eukaryotic translation initiation factor 4E binding protein), was hyper-phosphorylated when MYC was constitutively overexpressed. When the mTOR-mediated phosphorylation of 4EBP1 was blocked in MYC driven cancers by MLN0128, which specifically blocks the mTOR active site by inhibiting 4EBP1 phosphorylation, it specifically induced significant apoptosis in the MYC driven pre-tumor B cells, while sparing the wild type B cells. Their results suggest that, due to the synthetic lethality caused by specific inhibition of 4EBP1 in MYC-driven lymphomas and myelomas, a window of opportunity for therapeutic interventions can be created.

Other studies that have shown induction of synthetic lethality in MYC-driven tumors include use of small molecule inhibitors against Aurora kinases (AUK-A/B) [102], Bromodomain-containing 4 (Brd4) [103], Chk1 [104,105], MCL-1 [106], and Pim1/2 kinase [107,108].

#### E. CREB Binding Protein (CBP) and p300

Chromatin regulators, such as histone acetyl transferases (HAT), provide opportunities to exploit the differences between normal cells and the tumor cells. Many cancers, including non-small cell lung cancer, lymphomas, leukemia, and bladder carcinoma, exhibit a loss-of-function mutation in CBP, thus showing the crucial function of these epigenetic regulators. Targeting paralogs, where the cancer cells deficient in one gene show higher reliance on the other paralog, has proven to be successful in several instances [109]. Recently, a novel synthetic lethal relationship between the histone acetyl CBP and its paralog p300 was reported by Ogiwara and colleagues [110]. Their study shows that tumor cells that lack functional CBP can be specifically targeted by genetic or chemical inhibition of p300. The two histone acetylases have the common function of acetylating lysine K18 and K27 of histone H3 [110], thus acting as co-activators for many DNA binding transcription factors, such as MYC. In this study, the authors show that cancer knocking down p300 in CBP-deficient cells resulted in significant apoptosis as well as G1/S cell cycle arrest. A substantial reduction in MYC expression was observed in these cells, which can be attributed to the loss of acetylation on H3K27 and H3K18 in the *myc* promoter. Consequently, exogenous expression of MYC could rescue the synthetic lethality caused by p300 and CBP, suggesting MYC-dependent vulnerability when one of the paralogs were inactive or lost.

#### F. B-Cell Lymphoma 2 (Bcl-2) Small Molecule Inhibitors

Aberrant expression of Bcl-2 can help cancer cells by avoiding apoptosis. An increasing number of a diverse class of small molecule inhibitors (SMIs) of Bcl-2 can be specifically used for their therapeutic potential by inducing synthetic lethality in tumor cells with other oncogenic driver mutations. Bcl-2 can sequester pro-apoptotic proteins like Bid and Bim. By binding to the BH3 binding pocket, SMIs can act as BH3-mimetics, appropriating the sequestration, thus freeing the pro-apoptotic proteins. Several of these inhibitors, like ABT-737, ABT-263, ABT-199, Obatoclax, Gossypol, Apo-G2, etc., are currently in preclinical or clinical trial [111].

As mentioned earlier (under sub-section B), Corcoran and colleagues used a synthetic lethal screen in KRAS mutant tumors to find that ABT-263, in conjunction with MEK inhibitors, synergistically induced significant inhibition to of tumor growth [89]. This preclinical study has led to a clinical trial using ABT-263 and MEK inhibitor, Trametinib, in *kras* mutant tumors.

A more recent study by Chan et al. [112] identified cells with mutation in the isocitrate dehydrogenase (IDH) genes, in acute myeloid leukemia (AML), to be dependent on Bcl-2. IDH1 and IDH2 are crucial catalyzers in the conversion of isocitrate to a-ketoglutarate and their mutant forms lead to the production of the oncometabolite 2-HG, ultimately weakening the mitochondrial electron transport chain. On the other hand, Bcl-2 inhibits Bax, which can help increase mitochondrial membrane potential. Thus, cells with mutant IDH1 and IDH2 are significantly more sensitive to apoptosis when Bcl-2 is inhibited, compared to their wild type counterpart. Therefore, a synthetic lethal relationship can be exploited in these cells with therapeutic potential by use of small molecule inhibitors towards BCL-2, such as ABT-199.

Taken together, the studies support the possibility of therapeutic potential in using Bcl-2 inhibitors in susceptible tumors with other oncogenic driver mutations and/or in combination with MEK, ERK, or RAF inhibitors that can specifically target tumor cells.

#### G. Tumor Suppressor Gene, *p53*

Loss of TP53 has been implicated in many tumors, and besides its role in DNA damage response, when overexpressed, TP53 can induce apoptosis in some tumors, such myeloid leukemia cells. This suggests that TP53 also has a role in cell survival directly or indirectly [113]. Recent studies have also shown that TP53 can be activated by hypoxia and certain oncogenes to induce apoptosis [37,114]. In some *p53* null models, expression of wild-type p53 can lead to onset of apoptosis or induce senescence [115,116]. Mutations in *p53* can either be a loss-of-function or a gain-of-function. Cells that show a loss-of-function would lose their G1 checkpoint and be completely dependent on the G2/M checkpoint. This would make them more vulnerable to genotoxic reagents as well as ionizing radiation. In recent years, reports of several small molecule inhibitors have emerged that can induce synthetic lethality in p53 mutant background. Inhibitors of the G2/M checkpoint, such as UCN01 (Chk1 inhibitor), MK1775 (wee1 inhibitor), BI-2536 (PLK1 inhibitor), and PD0166285 (wee1 inhibitor) have been reported to cause apoptosis by synergism in p53 mutated cells, while sparing the normal cells [117,118,119]. As a caveat to exploiting the loss of p53 status, one needs to keep in mind the fact that under certain conditions, p53 can adopt a transient conformation and can in fact augment invasion and metastasis. In a study, it was reported that interaction of wild type p53 with the cytosolic chaperone protein, CCT, can promote p53 folding and in the absence of CCT, misfolded p53 accumulates. Interestingly, mutated p53 that cannot bind to CCT can undergo transient and unstable conformational change, resulting in promotion of invasion and metastasis at the same level as that of the wild type version [120]. Hence, the context of the cell is extremely important in designing such synthetic lethal combinations.

### 5.2. Necroptosis as a Therapeutic Weapon for Apoptosis-Resistant Cancer

Through the decades, many drugs have been successful in eradicating certain types of cancers. Most of these compounds were designed as pro-apoptotic therapy, since inducing apoptosis has been considered to be the principal method for cancer treatment. However, the efficacy of this therapy is limited by drug resistance. There are several factors that lead to drug resistance, such as disrupted apoptosis machinery, overactive pro-survival signaling pathways, increased expression of the therapeutic target, activation of alternative compensatory pathways, a high degree of molecular heterogeneity in tumor cells, upregulation of drug transporters, and multidrug resistance [59,121]. Among these findings, dysfunction of apoptosis appears to be a critical factor in intrinsic and acquired chemotherapeutic drug resistance. In cancer cells, genetic mutations and abnormal gene expression are widespread in the extrinsic and intrinsic apoptotic pathways, as previous studies have shown with increased expressions of anti-apoptotic proteins FLIP, Bcl-2, Bcl-_X_L, or MCL-1 and with mutations in p53, APAF-1, Bax, Fas, FADD, or caspases [59,121].

Since necroptotic and apoptotic pathway use different set of components, cancer cells, that are resistant to apoptosis-inducing agents, may be sensitive to necroptosis-inducing agents. This is because caspase activation, such as that of caspase-8, is required for apoptotic cell death, but necroptosis can eliminate cancer cells when caspase-8 is silenced, inhibited, or mutated. Therefore, necroptosis-based cancer therapy might be a novel alternative way of tackling apoptosis-resistant cancer cells [3,59]. This therapeutic strategy has been very recently proposed, but there are several issues that need to be addressed first.

The first concern involves the cancer cell’s ability to evade necroptosis when RIP1, RIP3, or MLKL are down-regulated or mutated. Therefore, it is essential to conduct a genetic screening to detect RIP1, RIP3, and MLKL status in patient tumor samples before using a necroptosis-inducing drug. If there are any abnormal or disrupted functions of necroptotic machinery, necroptosis-based therapy should not be chosen as the first line. However, there are strategies to increase the effectiveness of necroptosis-based therapy in necroptosis-resistant cancers. A recent study showed that RIP3 protein levels were increased after human colon cancer cells were exposed to a thermal dose, implying that the combination of heat therapy with necroptosis-based therapy may enhance the treatment efficacy [122]. Another strategy involves bypassing RIP1 to directly target RIP3, or bypassing RIP1 and RIP3 altogether, to directly target MLKL. For example, poly (I:C) can induce necroptosis in murine embryonic fibroblasts or human cervical cancer cells in a RIP1-independent manner. In addition, it was also reported that this RIP3-dependent necroptosis can promote the activation of dendritic cells to produce interleukin-12 (IL-12), which is critical for antitumor immunity [123], and the necroptotic cells may recruit monocytes, neutrophils, or macrophages to produce pro-inflammatory cytokines and to upregulate costimulatory molecules. Thus, triggering necroptosis might be a novel strategy for amplifying anti-tumor immunity [124]. Overcoming necroptosis resistance in cancer cells may be possible by successfully utilizing such strategies.

A second concern is the possibility of “off-target” effects by inducing necroptosis in normal cells and pathogenesis in some inflammatory diseases, such as inflammatory bowel diseases, Crohn’s disease, and inflammatory skin diseases [119]. This inflammation is reported to be induced by rapid and massive release of damage-associated molecular patterns, such as IL-1 family cytokines, nucleic acids, ribonucleoproteins, histones, HMGB family members, and heat-shock proteins [125]. From a safety standpoint, additional studies to evaluate other “off-target” effects of necroptosis-inducing agents are required.

## 6. Conclusions

The concept of cell death has evolved over time, necrosis being the oldest. In 1972, the concept of apoptosis was proposed and since then, this type of programmed cell death has been extensively studied. There was a long period of time when apoptosis was believed to be the only form of programmed cell death. However, several gaps in our understanding emerged, as many features of cell death could not be fully explained by necrosis and apoptosis. Degterev et al. first introduced the concept of necroptosis in 2005 [54], and since then, many characteristics of necroptosis have been discovered. Today, next to apoptosis, necroptosis has become one of the most explored mechanisms of programmed cell death. Other forms of programmed cell death, such as autophagy, pyronecrosis, pyroptosis, ferroptosis, and oxytosis have been examined [59,126] and while each of these processes have unique features, among them also exists substantial crosstalk and interdependent relationships. For the purpose of this review, we focused mainly on apoptosis and necroptosis, and on alternative cancer treatment options exploiting specific features of these processes. Although apoptosis was discovered many decades ago, we have only recently begun to learn the many apoptosis-related genes, which can be potential therapeutic targets. On the contrary, the era of necroptosis is still in its infancy, and clearly, more specific markers for necroptosis are needed. In addition, more extensive analysis of necroptosis, especially in vivo and in human tumor samples, will be necessary to make comparisons with apoptosis.

The idea of synthetic lethal interaction is very attractive since it kills the tumor cells specifically, while sparing the normal cells. It also allows targeting of those mutations/genes that are not easily “druggable” or tumors that are driven by loss-of-function mutations. It is certain that the modes of apoptotic and necroptotic cell death are mutually exclusive, but each of them can be used as an alternative weapon for killing cancer cells. To reach the full potential of this approach, further investigations and implementation of the most current technology is required. Bioinformatics and computational approaches using genome-wide interference studies, vulnerability screenings, CRISPR libraries, and other strategies can be powerful tools in this regard. With the help of these most modern tools, we can aim for a breakthrough by exploiting cancer’s Achilles’ heel.

## Figures and Tables

**Table 1:** Comparison of the features of apoptosis and necroptosis.

Features	Apoptosis	Necroptosis
Membrane blebbing	Yes	No
Cytoplasmic shrinkage	Yes	No
Apoptotic body	Yes	No
Nuclear fragmentation	Yes	No
Chromatin condensation	Yes	No
Swollen organelle and mitochondria	No	Yes
Membrane permeabilization	No	Yes
Caspase activation	Yes	No
RIP1/RIP3/MLKL activation	No	Yes
ROS production	Yes	Yes
ATP depletion	No	Yes
ATP increase	Yes	No
Executioner of cell death	caspase-3, caspase-7	ROS, homotrimerized MLKL
Selective inhibitor of cell death	zVAD (caspase inhibitor)	Nec-1 (RIP1 inhibitor)
		GSK-840/-843/-872 (RIP3 inhibitor)
		NSA (MLKL inhibitor)
